# Use of panitumumab in the treatment of acinar cell carcinoma of the pancreas: A case report

**DOI:** 10.3892/ol.2012.1083

**Published:** 2012-12-18

**Authors:** MANUEL MORALES, MIGUEL ÁNGEL CABRERA, MARIA DEL CARMEN MAESO, NOEMÍ FERRER-LÓPEZ

**Affiliations:** 1 Service of Medical Oncology, University Hospital Nuestra Señora de Candelaria, Santa Cruz de Tenerife, Canary Islands 38010, Spain; 2 Pathology, University Hospital Nuestra Señora de Candelaria, Santa Cruz de Tenerife, Canary Islands 38010, Spain

**Keywords:** acinar cell carcinoma of pancreas, panitumumab, KRAS

## Abstract

Two cases of stage IV acinar carcinoma of the pancreas are presented. The two patients were treated with several lines of chemotherapies active against colon cancer. At last-line, both patients received panitumumab monotherapy. We describe the tumour response to the different therapies. Our findings demonstrate that panitumumab produces objective responses when used as third-line treatment in the therapy of patients with acinar cell carcinoma of the pancreas. Thus, we propose the consideration of the use of panitumumab in early lines of treatment.

## Introduction

Acinar cell carcinoma (ACC) of the pancreas is a rare tumour, accounting for only 1% of cases of exocrine pancreatic malignancies. For patients in whom surgery with curative intent is not possible, there are no clear treatment guidelines (1). This tumour expresses molecular and genetic alterations characteristic of colon cancer (2). ACC has none of the gene abnormalities found in ductal pancreatic adenocarcinoma. In the current study we present two cases, initially treated with the same chemotherapy. The first patient responded to the initial treatment for some months, but failed the second and third line of chemotherapy. The second patient failed the first and second chemotherapy lines. Both patients received panitumumab monotherapy following the chemotherapy. The study was approved by the ethics committee of the University Hospital Nuestra Señora de Candelaria, Santa Cruz de Tenerife, Canary Islands, Spain. Written informed patient consent was obtained from the patients.

## Case reports

### Case 1

**Figure 1:**
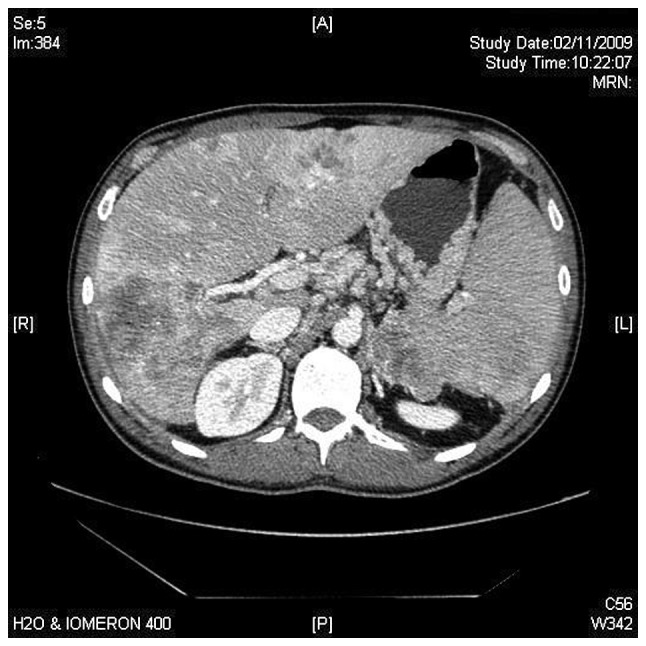
Abdominal CT images of patient 1 at diagnosis.

A 42-year-old male patient, with a previous history of asthma and type 2 diabetes mellitus (diagnosed 1 year before) and who was a past smoker, was admitted in November 2009 due to anorexia, weight loss (5 kg in the last 3 months), asthenia, abdominal pain and constipation. The physical examination revealed an oriented patient; no nodes were found; the lung and heart sounds were clear; the liver was enlarged (4 cm below the right costal margin); the spleen was felt 2 cm below the left costal margin; no oedema was present and the neurological examination was normal. The following tests were performed: i) Blood analysis and chemistry tests. The most notable reults were Hb 12 g/dl; leukocytes 5,400/mm^3^; platelets 173,000/mm^3^; glucose 113 mg/l; calcium 15 mg/dl; gamma glutamyl transpeptidase 177 U/l; alkaline phosphatase 149 U/l and α-fetoprotein (AFP) 16,7 ng/ml; other tests were normal; ii) CT of thorax and abdomen revealed bilateral pulmonary nodules of ∼1 cm in diameter and mediastinal lymph nodes, ranging between 1.1 and 2.1 cm. Multiple heterogeneous, ill-defined liver nodules of various sizes were visible (the largest being 8.8 cm diameter in the right lobe). There was also a pancreatic mass involving corpus and tail of 8×7.2 cm, infiltrating the left adrenal gland and spleen vessels. Spleen enlargement of 17.1 cm was noted (Fig. 1); iii) Percutaneous liver biopsy. The pathologic examination revealed the presence of an acinar cell carcinoma of the pancreas. The mutational state of KRAS was determined and tumour was *KRAS* wild-type.

**Figure 2:**
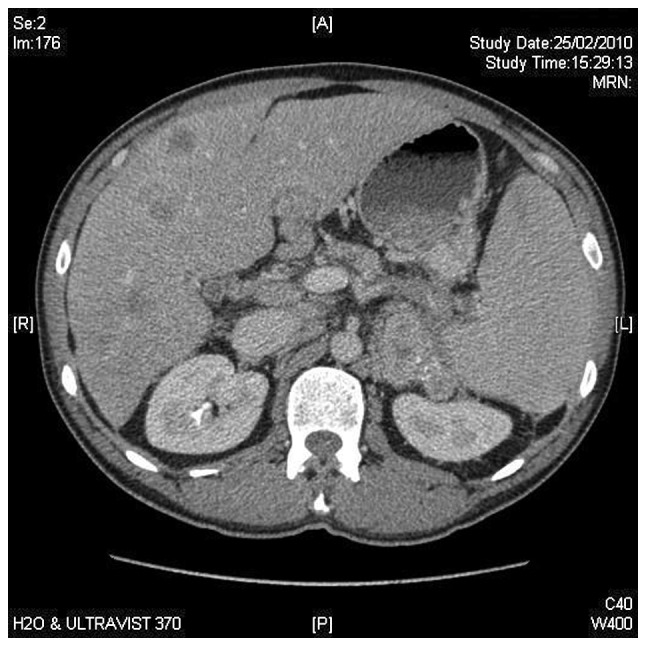
Abdominal CT images of patient 1, after two months of capecitabine/oxaliplatin therapy.

Calcium levels were controlled with i.v. saline/furosemide and zoledronic acid. Chemotherapy (capecitabine/oxaliplatin) was started. After three months of treatment, clinical and radiological improvement was achieved (Fig. 2). After the eighth cycle, the patient developed an allergy against oxaliplatin, and oxaliplatin was altered to irinotecan. In July 2010, progressive disease with refractory hypercalcaemia was diagnosed. Elevated levels of parathyroid hormone (PTH; 435.8 pg/ml) were detected and primary hyperparathyrodism was suspected. As magnetic resonance imaging (MRI) and sonography excluded the presence of parathyroid gland hyperplasia or tumour, an ectopic PTH secretion was suspected. Immunohistochemical study of the small amounts of tissue remaining from the percutaneous liver biopsy showed no evidence of PTH.

Thereafter, a third line of chemotherapy was started with bevacizumab-FOLFIRI. After 2 months of treatment, control of hypercalcaemia was achieved, with a drop in AFP levels. Nevertheless, the patient developed grade III gastrointestinal toxicity and persistent thrombopaenia. Monotherapy with panitumumab was started. After 3 doses, despite an improvement in liver function tests and AFP, the patient developed abdominal pain, delirium, significant asthenia and uncontrolled hypercalcaemia. Palliative treatment was started. The patient succumbed to the disease two days later, 12 months after diagnosis.

### Case 2

A 64-year-old male patient with a previous history of amoxycillin allergy, heavy smoking, chronic obstructive lung disease, alcohol intake (>40 g/day) and newly diagnosed type 2 diabetes mellitus, was admitted in December 2009 due to weight loss, fever and jaundice. The physical examination showed an oriented jaundiced patient, no nodes were felt, lung and heart sounds were clear; the abdomen was normal and no oedema was present. The following tests were performed: i) Blood analysis and chemistries. Total bilirubin 5.57 mg/dl; direct bilirubin 5.11 mg/dl; ASAT 274 U/l; ALAT 265 U/l; GGT 1,306 U/l; AP 730 U/l; CA19.9, 267.9 ng/ml; ii) CT of abdomen. Dilatation of extra-hepatic bile ducts, with a diameter of 1.5 cm, produced by an heterogeneous enlargement of the head of pancreas. Liver cysts in the left lobe and solitary metastasis (3.2 cm in diameter) in the right lobe. Palliative derivative surgery was performed in November 2009 and a mass protruding the duodenum was removed. The pathological examination of the mass revealed an ACC of the pancreas. The level of blood lipase was 410 U/l and that of AFP was 2,225.8 ng/ml. The *KRAS* was wild-type.

Chemotherapy with capecitabine/oxaliplatin was started. After 3 cycles of chemotherapy (March 2010), an increase in AFP (13.356 ng/ml) was observed and liver and regional node progression was diagnosed. Second-line therapy with weekly irinotecan was performed. In June 2010, disease progression (AFP, 23.062 ng/ml) was diagnosed. Panitumumab therapy was started and the patient remained clinically stable with a initial drop in AFP level, until December 2010. Then, weight loss and asthenia developed. Complementary tests showed an increase of AFP (75.871 ng/ml), progression in the primary lesion and liver metastases. The patient was transferred to the palliative care unit, and succumbed to the disease in January 2011.

## Discussion

ACC is defined as a carcinoma exhibiting evidence of pancreatic enzyme production by the neoplastic cells and may secrete AFP, endocrine products or lipase into the circulation. Patients experience disorders of the parathyroid glands, diffuse subcutaneous nodules and sclerotic lesions in cancellous bone; the latter two findings are due to fat necrosis in the subcutaneous tissue and bone (2).These tumours have a mutation in the *APC* gene/β-catenin pathway, with a genetic progression similar to colon cancer. The molecular changes found in ACCs are the loss of heterozygosity at 1p, 5q25 at the *APC* locus, 9p21 at the p16 locus and 17p13 at the *p53* locus. *K-ras* mutations are not identified in ACC, by contrast, activating mutations in the *K-ras* proto-oncogene are found in almost all cases of pancreatic ductal adenocarcinoma, as well as in early precursor lesions termed pancreatic intraepithelial neoplasia (3,4). ACC has none of the gene abnormalities that are commonly found in ductal pancreatic adenocarcinomas, pancreatic endocrine tumours and pancreatoblastoma (3,5,6).

Patients who are able to undergo surgical resection have a median survival of 36 months. Surgical management is not curative in the majority of patients. There is a 72% rate of recurrent disease among patients who undergo a surgical resection, a number of whom experienced distant metastases as opposed to local recurrences (2). Patients with stage IV disease have a 5-year survival rate of 17.2% (7). On multivariate analysis, age <65 years, well-differentiated tumours and negative resection margins are independent prognostic factors for ACC (7).

For locally advanced or metastatic disease, chemotherapeutic agents used in the treatment of colorectal cancer may be effective in ACC of the pancreas due to the genetic alteration in the *APC*/β-catenin pathway noted in acinar cells of the pancreas. 5-Fluorouracil (5-FU) is the most commonly used agent. Other agents that have been used are gemcitabine, cisplatin, doxorubicin, irinotecan, oxaliplatin, docetaxel, capecitabine, 5-FU/leucovorin, erlotinb, sunitinb and sirolimus (1,8).

Our two patients received chemotherapy combinations for colorectal cancers and their survival was similar, with a short objective response in patient 1. In a study of genetic abnormalities using fluorescence *in situ* hybridation (FISH) in cells of five patients with ACC, one patient had a survival period of 3 years after diagnosis and treatment with capecitabine followed by FOLFOX (folinic acid/5-FU/oxaliplatin). This patient had normal results in thymidine phosphorilase (*TYMP*) and thymidylate synthetase (*TYMS*), perhaps partly explaining why the patient responded to 5-FU and capecitabine (9).

**Figure 3:**
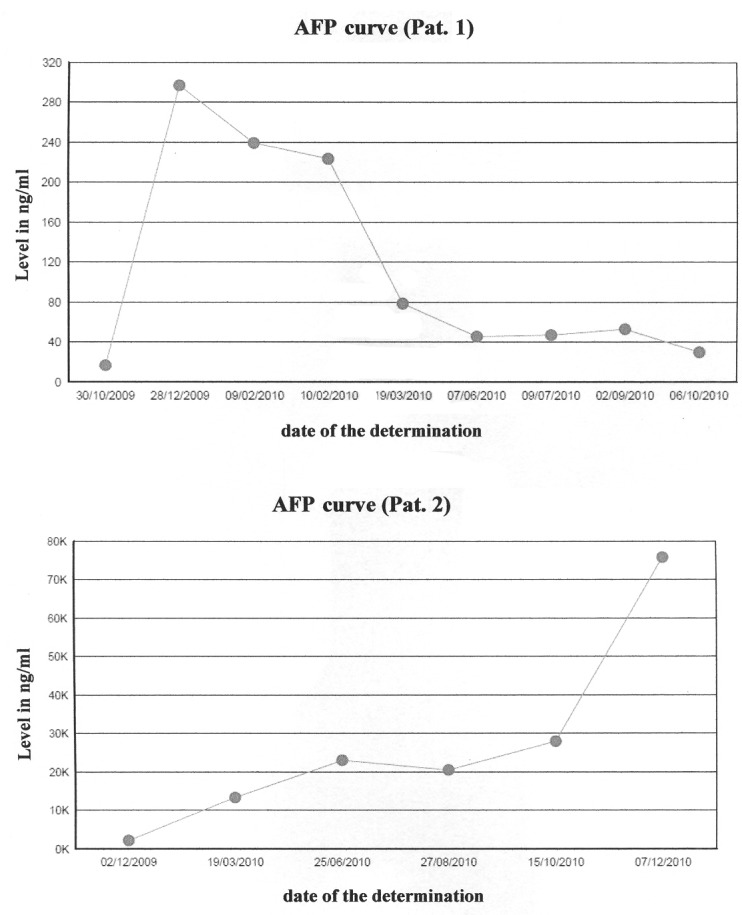
Evolution of AFP levels of both patients during therapy. AFP, α-fetoprotein.

Following the treatment guidelines for metastatic colorectal cancers expressing wild-type *K-ras* gene, anti-EGFR therapy with panitumumab was administered (10). With panitumumab, disease stabilisation for four months was achieved in patient 2, with a slight decrease in AFP levels. In patient 1, the three infususions of panitumumab produced an improvement in the liver function tests and AFP levels (Fig. 3). According to their molecular and genetic profiles, ACC may be tumours which benefit from targeted therapies.
